# Supplementary material for: Mutation-induced infections of phage-plasmids
Source: Nat Commun. 2023 Apr 12;14:2049. doi: 10.1038/s41467-023-37512-x (PMC10090143; doi:10.1038/s41467-023-37512-x)
Supplement: Supplementary file 3 — Description of Additional Supplementary Files [file 41467_2023_37512_MOESM3_ESM.pdf]

Description of Additional Supplementary Files:

Supplmentary data 1: Genes predicted on the phage-plasmid. Position on the phage-plasmid (Figure S4), translated protein sequences, transcripts per million (TPM) of RNA before or after the productive switch (Figure S6) are detailed. Genes homologous to the plasmid-related genes in the phage-plasmid in Denmark are marked with red (Figure 4). Genes homologous to the phage structural genes in the phage in the Arabian Sea are marked with yellow (Figure 4).
