# Supplementary material for: Mutation-induced infections of phage-plasmids
Source: Nat Commun. 2023 Apr 12;14:2049. doi: 10.1038/s41467-023-37512-x (PMC10090143; doi:10.1038/s41467-023-37512-x)
Supplement: Supplementary file 5 — Reporting Summary [file 41467_2023_37512_MOESM5_ESM.pdf]

nature portfolio

Corresponding author(s): Otto Cordero
Last updated by author(s): March 13, 2023

Reporting Summary

Nature Portfolio wishes to improve the reproducibility of the work that we publish. This form provides structure for consistency and transparency in reporting. For further information on Nature Portfolio policies, see our Editorial Policies and the Editorial Policy Checklist.

Statistics

For all statistical analyses, confirm that the following items are present in the figure legend, table legend, main text, or Methods section.

n/a	Confirmed	
☐	☒	The exact sample size (*n*) for each experimental group/condition, given as a discrete number and unit of measurement
☐	☒	A statement on whether measurements were taken from distinct samples or whether the same sample was measured repeatedly
☐	☒	The statistical test(s) used AND whether they are one- or two-sided<br>*Only common tests should be described solely by name; describe more complex techniques in the Methods section.*
☒	☐	A description of all covariates tested
☒	☐	A description of any assumptions or corrections, such as tests of normality and adjustment for multiple comparisons
☐	☒	A full description of the statistical parameters including central tendency (e.g. means) or other basic estimates (e.g. regression coefficient) AND variation (e.g. standard deviation) or associated estimates of uncertainty (e.g. confidence intervals)
☐	☒	For null hypothesis testing, the test statistic (e.g. *F*, *t*, *r*) with confidence intervals, effect sizes, degrees of freedom and *P* value noted<br>*Give P values as exact values whenever suitable.*
☒	☐	For Bayesian analysis, information on the choice of priors and Markov chain Monte Carlo settings
☒	☐	For hierarchical and complex designs, identification of the appropriate level for tests and full reporting of outcomes
☒	☐	Estimates of effect sizes (e.g. Cohen's *d*, Pearson's *r*), indicating how they were calculated

Our web collection on statistics for biologists contains articles on many of the points above.

Software and code

Policy information about availability of computer code

Data collection	No software was used in data collection.
Data analysis	Minimap v2.17, BCFtools v1.13, Skewer v0.2.2, FastQC v0.11.9, Bowtie2 v2.2.6, SAMTools v1.3.1, HTSeq v0.11.3, DeSeq2 v1.34.0, eggnog mapper v2, VIBRANT v1.2.0, MMseqs v2, R v4.1.0, clinker v0.0.23, ggmap v3.0.0, prodigal v2.6.3, custom R codes https://github.com/Xiaoyu2425/PP (DOI: 10.5281/zenodo.7730856)

For manuscripts utilizing custom algorithms or software that are central to the research but not yet described in published literature, software must be made available to editors and reviewers. We strongly encourage code deposition in a community repository (e.g. GitHub). See the Nature Portfolio guidelines for submitting code & software for further information.

Data

Policy information about availability of data

All manuscripts must include a data availability statement. This statement should provide the following information, where applicable:
- Accession codes, unique identifiers, or web links for publicly available datasets
- A description of any restrictions on data availability
- For clinical datasets or third party data, please ensure that the statement adheres to our policy

The sequencing data generated from this study, including the closed genome of Tritonibacter mobilis A3R06, whole genome sequencing and transcriptomic sequences for experimental evolution populations have been deposited in NCBI under accession code PRJNA895449 [https://www.ncbi.nlm.nih.gov/bioproject/PRJNA895449]. Source data are provided with this paper.

Human research participants

Policy information about studies involving human research participants and Sex and Gender in Research.

Reporting on sex and gender	This information is not collected in this study.
Population characteristics	This information is not collected in this study.
Recruitment	This information is not collected in this study.
Ethics oversight	This information is not collected in this study.

Note that full information on the approval of the study protocol must also be provided in the manuscript.

Field-specific reporting

Please select the one below that is the best fit for your research. If you are not sure, read the appropriate sections before making your selection.

☐ Life sciences ☐ Behavioural & social sciences ☒ Ecological, evolutionary & environmental sciences

For a reference copy of the document with all sections, see nature.com/documents/nr-reporting-summary-flat.pdf

Ecological, evolutionary & environmental sciences study design

All studies must disclose on these points even when the disclosure is negative.

Study description	This work studies experimental evolution of a phage-plasmid in the lab.
Research sample	The research sample is a marine bacteria, Tritonibacter mobilis A3R06, isolated from marine particles from coastal ocean water of Massachusetts, USA. The organism is studied for its global prevalence in the ocean ecosystem. This organism carries a phage-plasmid naturally, without any artificial manipulation.
Sampling strategy	Sampling was performed for the purpose of genome/transcriptome sequencing. 500uL of liquid bacterial culture is sampled to extract sufficient amount of DNA or RNA for Illumina sequencing. The liquid culture was well mixed before sampling.
Data collection	Data collection for culture growth was performed with a GENESYS 30 spectrophotometer, with optical density values recorded on lab notebook. Sequencing data was collected from the sequencing company, Microbial Genome Sequencing Center (Pittsburgh, PA).
Timing and spatial scale	Sampling of experimental evolution was from December 7th to December 14th, with liquid bacterial culture sampled every 24 hours from independent experimental evolution populations of Tritonibacter mobilis A3R06. All culture was in 15 mL test tubes in the lab, which marks the spatial scale of the sampling efforts.
Data exclusions	No data is excluded from analyses.
Reproducibility	Experimental evolution was performed in 15 independent replicate lines to ensure reproducibility.
Randomization	This is not relevant to the study as no group was applied.
Blinding	This is not relevant to the study as the sampling was from completely-mixed liquid culture of bacteria.

Did the study involve field work? ☐ Yes ☒ No

Reporting for specific materials, systems and methods

We require information from authors about some types of materials, experimental systems and methods used in many studies. Here, indicate whether each material, system or method listed is relevant to your study. If you are not sure if a list item applies to your research, read the appropriate section before selecting a response.

Materials & experimental systems

n/a	Involved in the study
☒	☐ Antibodies
☒	☐ Eukaryotic cell lines
☒	☐ Palaeontology and archaeology
☒	☐ Animals and other organisms
☒	☐ Clinical data
☒	☐ Dual use research of concern

Methods

n/a	Involved in the study
☒	☐ ChIP-seq
☒	☐ Flow cytometry
☒	☐ MRI-based neuroimaging
